# Viral Interference with DNA Repair by Targeting of the Single-Stranded DNA Binding Protein RPA

**DOI:** 10.1371/journal.ppat.1003725

**Published:** 2013-10-24

**Authors:** Pubali Banerjee, Rowena deJesus, Ole Gjoerup, Brian S. Schaffhausen

**Affiliations:** 1 Department of Developmental, Molecular and Chemical Biology, Tufts University School of Medicine, Boston, Massachusetts, United States of America; 2 Program in Molecular Microbiology, Sackler School of Graduate Biomedical Sciences, Tufts University School of Medicine, Boston, Massachusetts, United States of America; 3 Molecular Oncology Research Institute, Tufts Medical Center, Boston, Massachusetts, United States of America; University of Michigan, United States of America

## Abstract

Correct repair of damaged DNA is critical for genomic integrity. Deficiencies in DNA repair are linked with human cancer. Here we report a novel mechanism by which a virus manipulates DNA damage responses. Infection with murine polyomavirus sensitizes cells to DNA damage by UV and etoposide. Polyomavirus large T antigen (LT) alone is sufficient to sensitize cells 100 fold to UV and other kinds of DNA damage. This results in activated stress responses and apoptosis. Genetic analysis shows that LT sensitizes via the binding of its origin-binding domain (OBD) to the single-stranded DNA binding protein replication protein A (RPA). Overexpression of RPA protects cells expressing OBD from damage, and knockdown of RPA mimics the LT phenotype. LT prevents recruitment of RPA to nuclear foci after DNA damage. This leads to failure to recruit repair proteins such as Rad51 or Rad9, explaining why LT prevents repair of double strand DNA breaks by homologous recombination. A targeted intervention directed at RPA based on this viral mechanism could be useful in circumventing the resistance of cancer cells to therapy.

## Author Summary

DNA repair protects genome integrity and unrepaired DNA damage can cause cancer. We have identified a new mechanism by which a tumor virus makes cells hypersensitive to DNA damage. The Large T Antigen (LT) of polyoma virus blocks DNA repair pathways, making cells 100 fold more sensitive to DNA damage. LT does this by targeting replication protein A (RPA). RPA is central to both DNA replication and repair. Ordinarily RPA and then other DNA repair proteins are recruited to sites of DNA damage. LT blocks recruitment of these proteins to damage foci. Current cancer treatment strategies like radiation therapy and chemotherapeutics cause DNA damage to block the growth and spread of cancer. This work suggests a target that might increase the efficacy of such treatment.

## Introduction

Because genomes are subject to different kinds of insults, cells have evolved a variety of mechanisms to repair damage [1]. Homologous recombination (HR), non-homologous end joining (NHEJ), base excision repair (BER), nucleotide excision repair (NER), and mismatch repair (MMR) are repair systems designed to counter different kinds of damage. Inability to correct nascent mutations is an important issue in cancer. Estimates suggest that there are from 1,000 up to 100,000 somatic mutations in common adult cancers [2].

DNA viruses have discovered the value of manipulating DNA repair pathways [3]. ATM, which is activated at double-strand breaks (DSBs) [4], is associated with replication of viruses like SV40, murine polyomavirus, herpes simplex virus (HSV), human cytomegalovirus (HCMV), and Epstein Barr virus (EBV) [3]. For murine polyoma, replication is tenfold less efficient in ATM (−/−) fibroblasts than in wild type cells [5]. The DNA damage response contributes to SV40 DNA replication [5], [6], [7]. ATM phosphorylation of SV40 LT antigen is important for viral DNA synthesis [3]. A decrease in ATM function reduces SV40 DNA synthesis postponing both formation of viral replication centers and recruitment of DNA repair proteins at these sites [3]. Activation of ATM and the MRN (MRE11/Rad50/NBS1) complex regulates HSV-1 replication. However, adenovirus (Ad) specifically inactivates the MRN complex by either mislocalization or degradation at the infection onset to promote Ad DNA replication [8]. SV40 LT deregulates multiple DNA damage pathways [4]. SV40 LT forms a tight complex with NBS1, one member of the MRN complex [9]. Levels of MRN subunits decline during SV40 infection [10]. SV40LT expression induces promyelocytic leukemia protein interaction with RAD51 [4].

Although different kinds of repair mechanisms, each constituting a complex network of signaling components, coordinate responses to different kinds of DNA damage, a common molecular component that responds to most genotoxic insult is RPA [11]. RPA has been shown to be involved in both repair of UV damage [12] and MRN complex recruitment to DSBs induced by etoposide [13]. RPA acts as a sensor for UV induced DNA damage that recognizes cyclobutane thymine dimers and regulates the efficient removal of the lesion [14]. In addition, it participates in the formation of repair foci in response to etoposide induced DSBs [13]. Furthermore, depletion of RPA has been shown to cause spontaneous DNA damage and apoptosis in HeLa cells [15]. ATM can phosphorylate RPA [16], [17]. This is an example of cross talk among the repair proteins and underscores the complexity of the DNA damage response (DDR).

Polyoma LT plays critical roles in the viral life cycle. Broadly, these can be divided into issues related to DNA replication or to control of cell phenotype. In productive infection, LT initiates viral DNA replication [18], has helicase [19] and ATPase activities [20] and associates with pol α-primase [21], as well as promotes integration of the viral genome into the host [22] or promotes recombination [23]. It has numerous effects on cell phenotype, many of which are dependent on its association with members of the retinoblastoma tumor suppressor family. For example, it immortalizes primary cells [24], blocks differentiation [25] and promotes apoptosis [26].

This work describes a new connection between DNA viruses and DNA repair pathways. Binding of RPA by LT sensitizes host cells to DNA damage by as much as 100-fold. Since the same result is obtained with UV irradiation or etoposide exposure, agents that cause different kinds of lesions, multiple repair systems are being affected. Mapping indicates that binding of the origin-binding domain (OBD) of LT to RPA is sufficient to sensitize cells. Confirming this connection, cells overexpressing RPA are protected from LT, while knockdown of RPA triggers sensitization of cells when exposed to DNA damage even in the absence of LT. LT prevents the recruitment of RPA to DNA damage repair foci, suggesting why repair fails.

## Results

### Murine Polyomavirus by the Action of Large T Antigen Sensitizes Cell to DNA Damage

**Figure 1:**
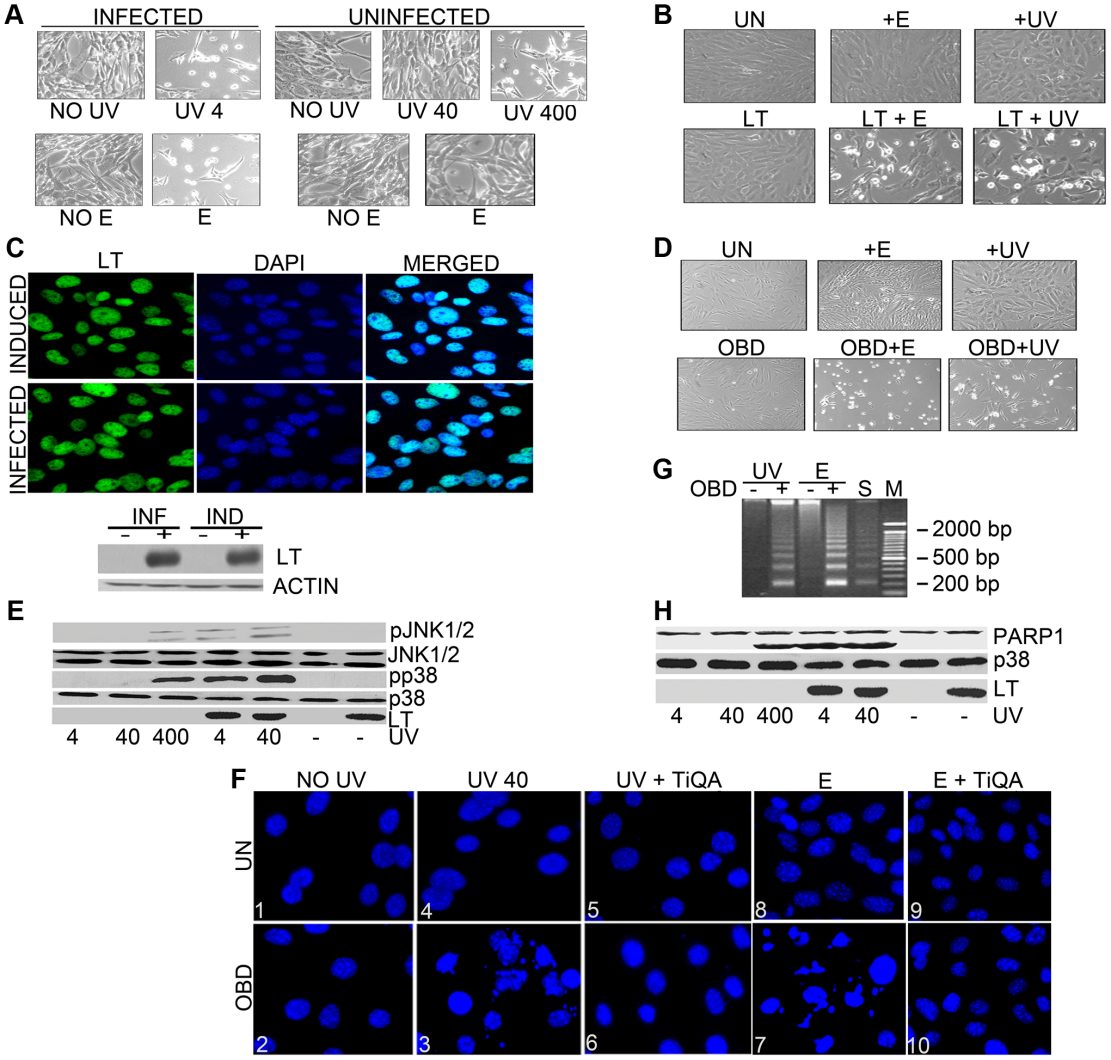
Polyoma large T sensitizes cells to DNA damaging agents. A : Secondary mouse embryo fibroblasts (MEFs) uninfected or infected were untreated (negative control) or exposed to UV light (4, 40, or 400 J/m^2^) or treated by addition of etoposide (100 µM) to the medium at 18 hours post infection. Phase contrast pictures were taken sixteen hours later. **B**: Mouse embryo fibroblasts (MEFs) uninduced (UN) or induced to express LT by the removal of doxycyclin for 48 hours were untreated, exposed to a 40 J/m^2^ dose of UV light or treated by addition of etoposide (100 µM) to the medium. Phase contrast pictures were taken sixteen hours later. **C: Top Panel**: MEFs expressing LT (48 hours post induction) or infected secondary MEFs (18 hours post infection) were stained with antibody to LT (FITC). Individual fluorescence images and the merged DAPI images that stain nuclei are shown. **C: Bottom Panel**: Cell extracts from uninduced MEFs or MEFs expressing LT (48 hour post-induction) and infected secondary MEFs (18 hours post infection) or uninfected MEFs were harvested, separated by SDS PAGE, and blotted with antibodies against LT or actin (loading control). **D**: Mouse embryo fibroblasts (MEFs) uninduced (UN) or induced to express OBD by the removal of doxycyclin for 48 hours were untreated (negative control), exposed to a 40 J/m^2^ dose of UV light or treated by addition of etoposide (100 µM) to the medium. Phase contrast pictures were taken sixteen hours later. **E**: Polyoma large T enhances stress responses to UV: Uninduced MEFs or those expressing LT were untreated or exposed to UV light (4, 40, or 400 J/m^2^), 48 hours post induction. (For LT-induced MEFs, no cells remain after 400 J/m^2^.) After one hour, cell extracts were harvested, separated by SDS PAGE, and blotted with antibodies against phospho JNK (pJNK1/2), total JNK, phospho p38 (pp38), total p38 or LT. **F**: DAPI staining of nuclear chromatin from uninduced (UN) or OBD-expressing cells 6 h after UV (0 and 40 J/m^2^) or etoposide treatment (100 µM) (E) in the presence or absence of overnight pretreatment with PARP inhibitor TiQA (30 mM). Panel 3 (OBD+ UV40) and panel 7 (OBD+ E) show apparent hallmarks of apoptosis with densely stained nuclear granular bodies within fragmented nuclei, highly condensed and fragmented chromatin. Panel 6 and panel 10 show lightly and evenly stained nuclei indicating that TiQA protects cells from apoptotic induction by UV irradiation and etoposide treatment. **G**: OBD enhances DNA laddering: Low molecular weight DNA was extracted from uninduced or OBD expressing MEFs after 40 J/m^2^ UV or 100 µM etoposide (E) treatment. Serum starved NIH 3T3 positive controls (S) cells undergoing apoptosis exhibit DNA laddering. Lane M represents DNA size markers. **H**: Cell extracts as in E were tested by western blotting for LT and PARP-1, with p38 as a loading control.

Infection with murine polyomavirus sensitized cells to DNA damage. Treatment of virus-infected secondary mouse embryo fibroblasts with 4 J/m^2^ dose of UV or 100 µM of etoposide at eighteen hours after infection led to rapid cell death as seen in the phase microscope at 24 hours after infection (Fig. 1A). By contrast, uninfected cells were not obviously affected by UV at 40 J/m^2^ or 100 µM of etoposide, presumably because they could repair the DNA damage. Killing of controls comparable to that seen in the infected cells was observed only at a much higher dose of UV (400 J/m^2^). This raised the possibility that polyomavirus was interfering with DNA repair. Since previous work indicated that SV40LT could interact with DNA repair proteins such as NBS1 [9] or RPA, we hypothesized that PyLT might be involved. To study whether polyoma LT affects cellular responses to DNA damage, immortalized mouse embryonic fibroblasts (MEFs) were prepared that conditionally expressed full-length LT using doxycycline in a tet-off system. Uninduced cells and cells expressing LT were treated with UV irradiation (40 J/m^2^) or with 100 µM etoposide. While UV light primarily causes photoproducts, etoposide induces strand breaks in DNA by inhibiting topoisomerase II. By 16 hours after DNA damage, MEFs expressing LT showed a dramatic change in phenotype (Fig. 1B). LT expressing cells exposed to UV-irradiation or etoposide looked rounded, refractile and displayed a loss of cell-to-cell contact. Uninduced mouse embryo fibroblasts exposed to these levels of damaging agents or LT-expressing cells not exposed to DNA damaging agents did not show these morphological changes. The expression of LT in infected cells and after induction in the inducible cell line was similar (Fig. 1C and Fig. S1). Immunofluorescence showed that in each case virtually all cells expressed LT, while western blotting of cell extracts showed that the levels of LT expression were similar. Because the LT origin-binding domain (OBD, LT residues 264–420) interacts with DNA, its role in sensitivity to damage was tested in cells conditionally expressing it (Supplemental Fig. S1). OBD induces dramatic changes in phenotype similar to full-length LT following UV-irradiation or etoposide treatment (Fig. 1D). In general, the effects on DDRs described here for full-length LT can be demonstrated with the OBD alone.

Several lines of evidence suggested that the cells were showing enhanced stress from DNA damage and were dying from apoptosis. Fig. 1E shows that LT-expressing cells have enhanced activation of JNK1 and 2 as well as p38 as determined by activation-specific phosphoantibodies after as little as 4 J/m^2^ UV treatments. In control cells, 400 J/m^2^ UV was required to produce the same activation as LT-expressing cells treated with 1/100 the dose. DAPI-staining of nuclear chromatin showed a large number of condensed and fractured nuclei in OBD- or LT- expressing cells following UV at 40 J/m^2^ and etoposide (100 µM) (Fig. 1F). DNA fragmentation, a characteristic marker of apoptosis, was seen by DNA laddering in the cells that express OBD post-UV irradiation or etoposide treatment (Fig. 1G). Another marker for apoptosis is the activation and cleavage of PARP (Poly ADP Ribose polymerase-1) [27]. LT enhanced the activation of poly ADP ribose polymerase (PARP) as seen by its cleavage (Fig. 1H). Again it took 100 times as much UV to generate the same amount of PARP cleavage in control cells as in LT expressing cells. Inhibition of PARP by pretreating cells overnight with a PARP inhibitor (30 µM of TiQA) prior to UV exposure had no effect on the early stress responses of Jnk and p38 activation (not shown). However, the activation of PARP was important for the apoptosis, because cells were protected against either UV or etoposide when pretreated overnight with 30 µM of TiQA (Fig. 1F, Panel 6 and 10).

**Figure 2:**
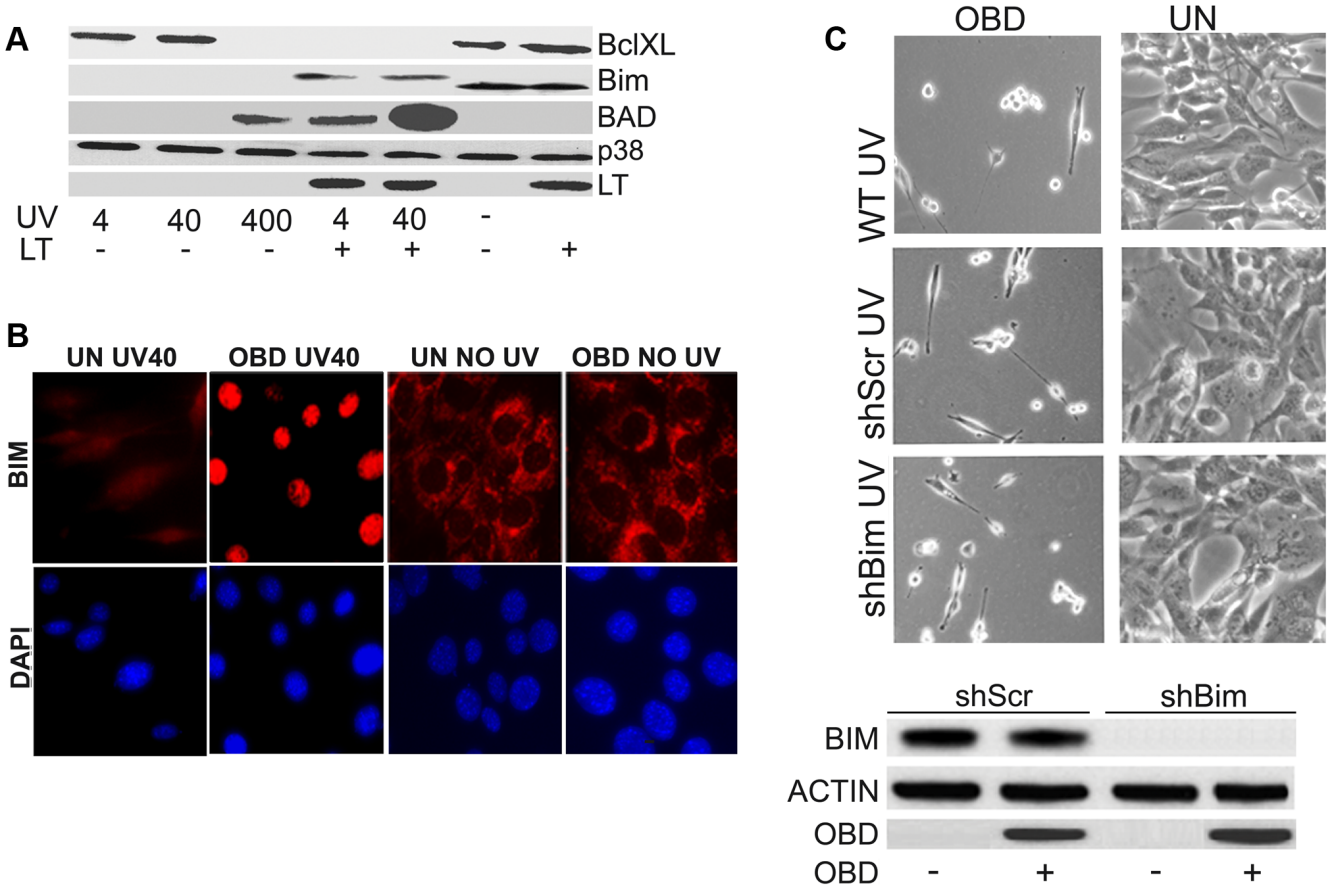
Sensitization by LT triggers apoptotic changes. A : Uninduced MEFs that were not expressing LT and LT-expressing MEFs were unexposed or exposed to increasing amounts of UV (4, 40 or 400 J/m^2^). After one hour, cell extracts were harvested, separated by SDS PAGE, and blotted with antibodies against BAD, BclXL, Bim, LT, with p38 as a loading control. **B**: OBD affects localization of Bim after UV exposure. Uninduced MEFs (UN) not expressing or MEFs expressing OBD were untreated or exposed to 40 J/m^2^ UV light. After one hour, they were stained (TRITC) with antibody to Bim and DAPI. Individual fluorescence images are shown. **C: Top Panel**: Morphologies of cells in which Bim has been knocked down. Stable MEF cell lines that inducibly express OBD were used to obtain cells in which Bim was stably knocked down using shRNA directed towards Bim. Morphologies of uninduced cells (UN) and cells expressing OBD are shown 16 hours after exposure to 40 J/m^2^ UV light. Scrambled shRNA (shScr) containing uninduced (UN) MEFs and OBD expressing MEFs without shRNA were used as controls. **Bottom Panel**: Expression in uninduced MEFs (UN) or MEFs expressing OBD with shRNA targeting Bim. Cell extracts harvested 1 h post treatment were resolved by SDS PAGE and tested by western blotting for Bim, OBD with actin as the loading control. Scrambled shRNA (shScr) MEFs and MEFs expressing OBD were used as negative controls.

Apoptotic cell death in response to UV [28] or etoposide [29] has been recognized for a long time. Changes in death proteins are expected in cells undergoing apoptosis. The pro-death protein BAD is upregulated in cells expressing LT treated with 4 J/m^2^ UV, but only in control cells when treated with 100 times the dose of UV (Fig. 2A). In parallel, the pro-survival protein BclXL is down regulated in LT expressing cells and uninduced cells treated with high levels of UV. There is one difference between LT expressing cells and controls. BIM, a proapoptotic BH3 protein of the Bcl2 family disappears after UV treatment even at low UV dose in controls while it is shifted in mobility, but only slightly decreased, in LT cells. Moreover, after UV treatment in OBD-cells, Bim unexpectedly translocates to the nucleus (Fig. 2B). The significance of this effect is unclear, because efficient knockdown of Bim did not protect cells from enhanced damage caused by OBD (Fig. 2C).

### Polyoma LT/OBD Enhances DNA Damage from UV Irradiation and Etoposide Treatment

**Figure 3:**
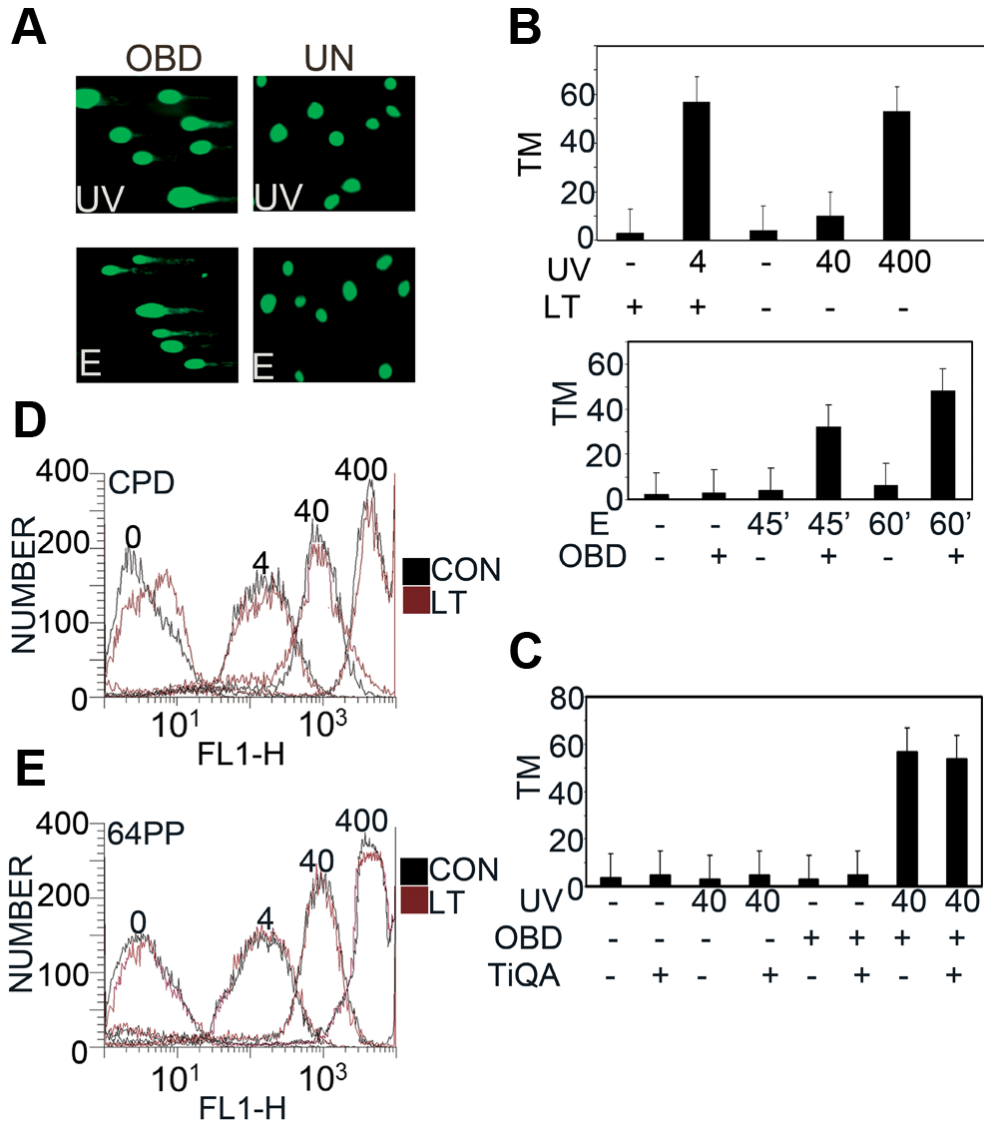
DNA damage after UV irradiation and etoposide in the presence of LT or OBD. A : Representative images of comet assays from uninduced MEF cells or OBD expressing cells after 40 J/m^2^ UV, or 100 µM etoposide (E). **B**: Top Panel: CASP calculated tail moments (TM) from analysis of comet assays of uninduced or LT expressing MEFs immediately after UV at 4, 40 or 400 J/m^2^. Data are shown for a representative experiment, where at least 100 comets were quantitated for each cell line. Bottom Panel: Uninduced or OBD-expressing MEFs were untreated or exposed to etoposide (E), 0 and 100 µM, for 45 or 60 minutes. CASP calculated tail moments (TM) from analysis of alkaline comet assays are shown. **C**: Quantification of DNA damage with or without TiQA (30 mM) pretreatment in uninduced or OBD expressing MEFs after exposure to 40 J/m^2^ UV Irradiation. CASP calculated tail moment (TM) from analysis of comet assays are shown for a representative experiment, where at least 100 comets were quantitated for each condition. **D, E**: LT/OBD Does Not Affect Formation of UV Photolesions. **D**: MEFs inducibly expressing LT or uninduced controls (CON) were exposed to 0, 4, 40 and 400 J/m^2^ UV irradiation and then stained immediately with antibody against CPD to measure the degree of damage by FACS analysis. **E**: As in D, except stained immediately with anti-64PP antibody.

Although LT effects on survival might arise by modulating survival pathways, it seemed more likely that LT was enhancing DNA damage. Comet assays can be used to detect DNA breaks in single cells [30]. Damage is seen as a comet that can be quantified by calculating tail moments that reflect the relative amount and distribution of DNA in the tail. MEF controls or cells induced to express OBD were exposed to UV light (40 J/m^2^) or etoposide (100 µM). Comet tails were observed for OBD (or LT) expressing cells that had been exposed to DNA damage (Fig. 3A). Cells that did not express LT or OBD displayed nuclear DNA without the characteristic streaming that is observed in the presence of DNA damage. Average tail moments can be calculated giving a quantitative estimate of damage [31]. In the experiment of Fig. 3B, the tail moment for LT went from 4 to 55 after UV treatment. Uninduced cells could be treated with UV to produce comets, but it again required much higher doses of UV (400 J/m^2^) to produce the same effect as LT at 4 J/m^2^. Etoposide treatment also resulted in more DNA breaks in OBD expressing cells than in uninduced cells.

A question might be whether DNA breakage seen in comet assays reflects apoptosis triggered by DNA damage treatments of cells expressing LT/OBD. Two kinds of observations argue against this. First, comet tails were observed even when cells were processed immediately after UV treatment. More convincingly, treatment with PARP inhibitor TiQA blocked death and nuclear fragmentation (Fig. 1F), but had no effect on the generation of comets immediately after UV treatment (Fig. 3C). Both results indicate that breakage is part of the DNA damage/repair process and not apoptosis.

The next question is whether the cells expressing LT are more sensitive to the initial DNA insult, perhaps from a change in chromatin structure, or whether the effect is more downstream, at the level of DNA repair. This is most easily tested after UV irradiation. Cyclobutane pyrimidine dimer (CPD) and pyrimidine-pyrimidone (6-4) photoproduct (64PP), the major DNA lesions directly induced by UV irradiation, are recognizable by antibodies against the altered bases [32]. FACS analysis shows that CPD formation increases as the dose of UV increases, but expression of LT has no effect (Fig. 3D). The same result is seen in Fig. 3E for 6-4 photoproducts. These experiments suggest that LT affects the repair process and not initial formation of damaged DNA.

### Genetic Analysis of OBD Function Shows Binding to RPA Is the Basis for Sensitization to DNA Damaging Agents

**Figure 4:**
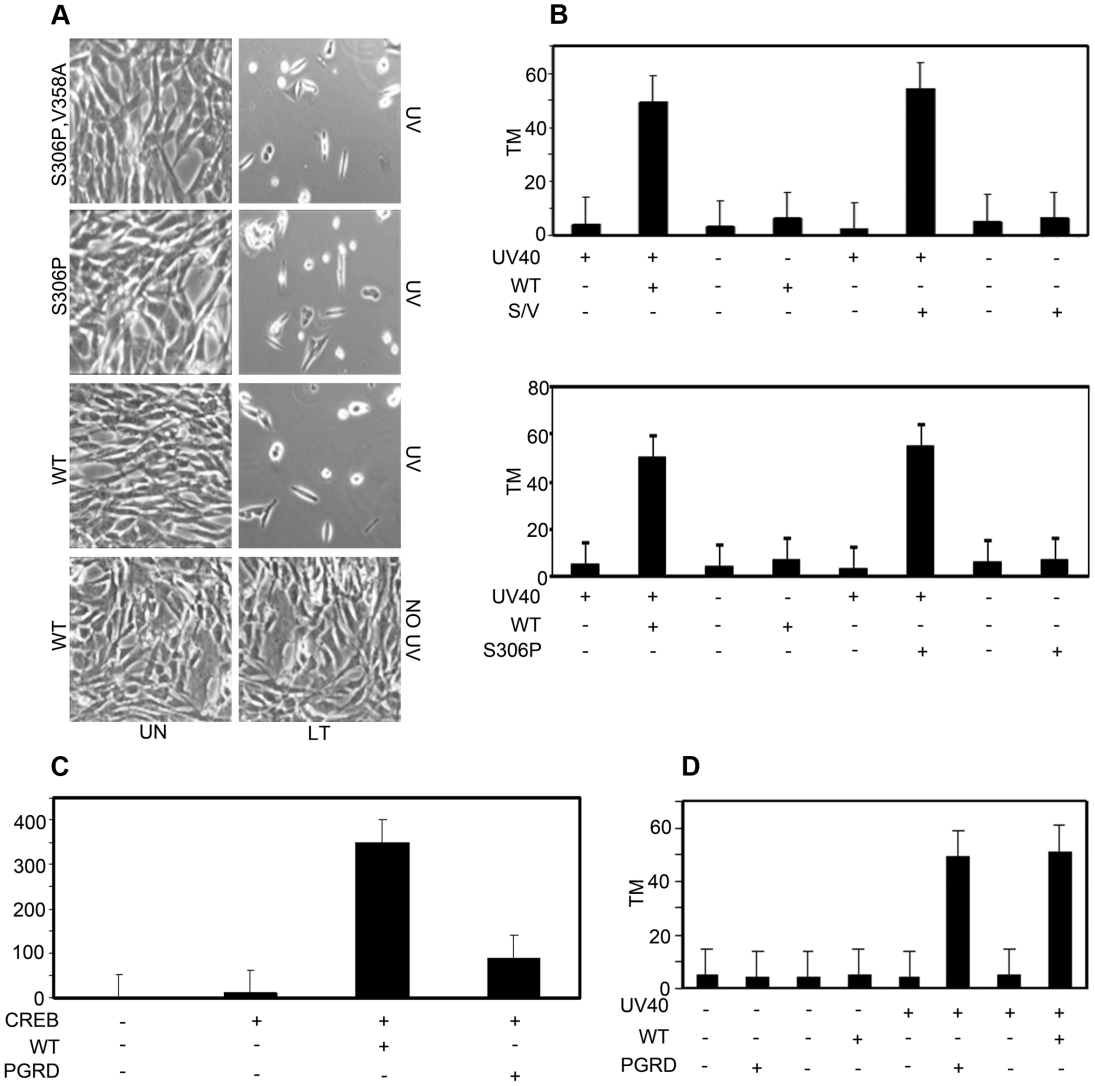
Neither DNA binding nor transcriptional activation are required for enhancement of DNA damage. A : Site-specific (S306P) and non-specific (S306P/V358A) DNA binding mutant of LT sensitize cells to UV. Uninduced (UN) MEFs or MEFs inducibly expressing wild type (WT) and S306P T or S306P,V358A T were untreated or exposed to UV light (40 J/m^2^). Morphology of uninduced cells (UN) and cells expressing OBD is shown 16 h after exposure to UV light (40 J/m^2^). **B**: Top Panel: CASP calculated tail moments (TM) from analysis of comet assays from uninduced MEFs, wild type (WT) or S306P,V358A (S/V) expressing cells that were untreated or treated with UV (40 J/m^2^). Data are shown for a representative experiment, where at least 100 comets were quantitated for each cell line. Bottom Panel: CASP calculated tail moment (TM) from analysis of comet assays from uninduced MEFs, wild type (WT) or S306P expressing cells that were untreated or treated with UV (40 J/m^2^). Data are shown for a representative experiment, where at least 100 comets were quantitated for each cell line. **C**: NIH 3T3 cells maintained under growing conditions (10% CS) were cotransfected with Gal4TK-Luc reporter and Gal4-CREB (CREB) and WT OBD or mutant P402R/G403D (PGRD). Cells were harvested 48 hours post-transfection and assayed for luciferase activity. Assays were done as previously described (30). **D**: CASP calculated tail moment (TM) from analysis of comet assays from uninduced MEFs, wild type (WT) or PGRD LT expressing cells that were untreated or treated with UV (40 J/m^2^) and immediately analyzed for comets. Data are shown for a representative experiment, where at least 100 comets were quantitated for each cell line.

The OBD of LT is multifunctional. It binds DNA specifically at GAGGC pentanucleotides and also binds DNA in a non-site-specific manner [33], [34]. The OBD activates transcription through CREB sites, in part by binding CREB [34]. Mutants defective in DNA-binding and activation of transcription sensitize cells to DNA damage just like wild type. Stable MEF cell lines that express mutant S306P defective for sequence specific recognition and the double mutant S306P/V358A defective even for non-specific DNA binding (Fig. S1) still caused the same sensitization to DNA damage seen by morphology and comet assay as wild type (Fig. 4A & 4B). Additionally, stable MEF cell lines that express mutant P402R/G403D (PGRD) and E343K/E344K (343KK) defective in transcriptional activation (Fig. S1) nonetheless sensitized cells. Mutant PGRD near the end of the OBD showed reduced transactivation of CREB responsive promoters (Fig. 4C). Comet assays confirmed a significant increase in DNA damage in MEFs expressing the mutant forms of LT (Fig. 4D). Neither DNA binding nor ability to activate transcription are therefore important for sensitization to damage.

Since LT sensitizes cells to different kinds of DNA damage, it is plausible that some element common to repair of different kinds of damage is targeted by OBD. RPA, a heterotrimeric, single-stranded DNA binding protein is such a protein [35]. Furthermore, RPA is an indispensable component of polyomavirus DNA replication [36], [37]. A physical interaction between SV40 LT and the RPA high-affinity ssDNA-binding domains was mapped to the SV40 OBD [38].

**Figure 5:**
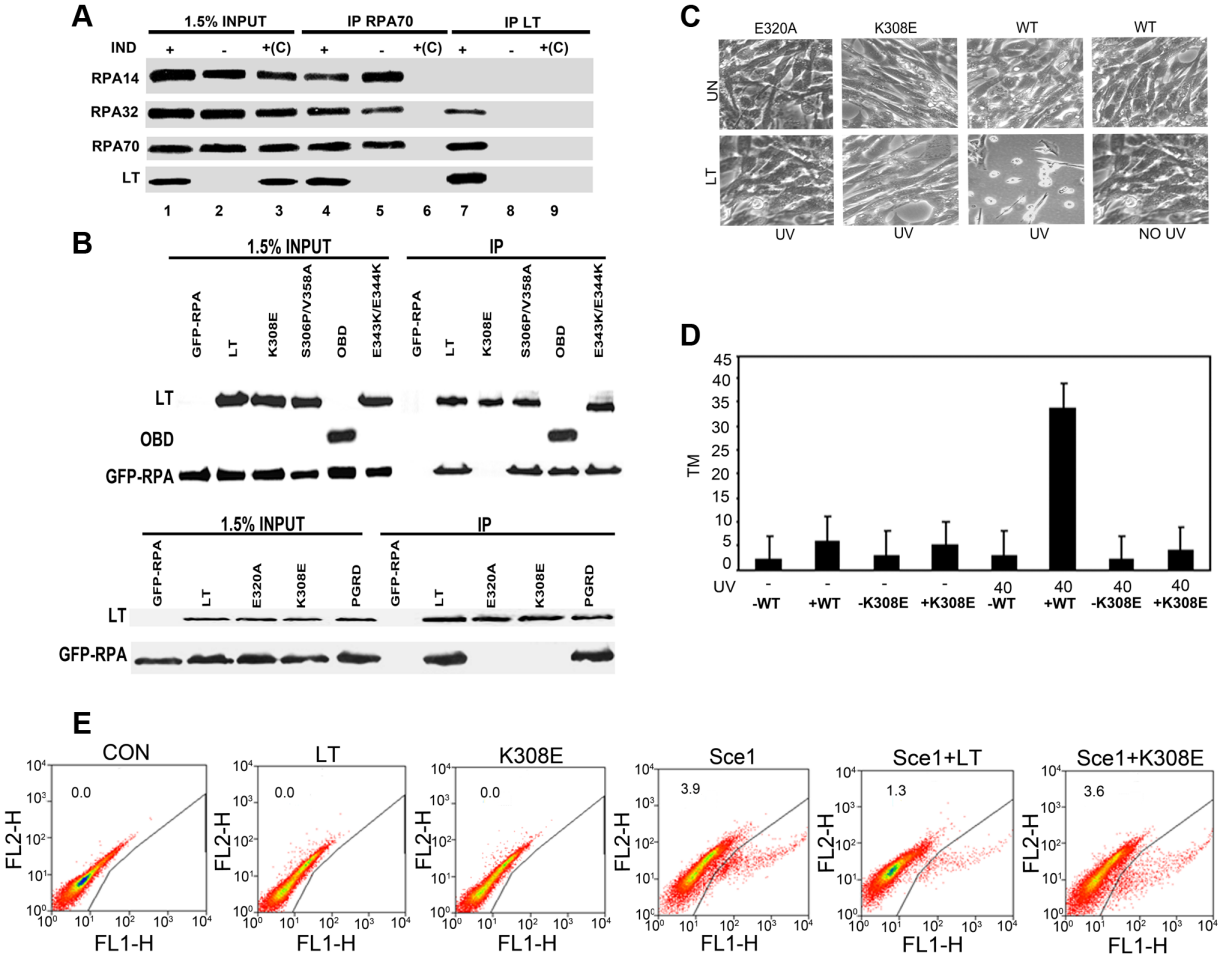
The binding of OBD/LT to RPA is necessary to sensitize cells to damage. A: Binding of RPA: Uninduced MEFs (IND −) or MEFs inducibly expressing wild type LT (IND +) for 48 h were harvested and then immunoprecipitated with anti-T or anti-RPA70. The immunoprecipitates and whole cell extracts were blotted with antibody against endogenous RPA14, RPA32, RPA70 and LT. Extracts with only agarose beads and no antibody were used as control +(C). **B**: 293T HEK cells were cotransfected with control CMV vector (−) or CMV vectors expressing wild type LT; S306P/V358A; E343K/E344K; HA-OBD and K308E (Top Panel) and wild type LT, E320A, K308E and PGRD (Bottom Panel) as well as GFP-tagged RPA. Cells were harvested 48 hours post transfection and then immunoprecipitated with anti-T or anti-HA serum (OBD). The immunoprecipitates and whole cell extracts were blotted with antibody against GFP to show RPA70, against LT and against HA to show OBD. **C**: RPA binding defective mutants K308E and E320A fail to sensitize cells to UV. MEFs inducibly expressing wild type, E320A, or K308E LT were untreated or exposed to UV light (40 J/m^2^). Morphologies of the cells are shown 16 hours after stress treatment. **D**: CASP calculated tail moments (TM) from analysis of comet assays from uninduced MEFs, wild type (WT) or K308E LT expressing cells that were untreated or treated with UV (40 J/m^2^) and immediately analyzed for comets. **E**: LT interferes with double-stranded DNA break repair: DR-U2OS cells maintained under growing conditions (10% FCS) were cotransfected with I-SceI plasmid and empty vector, LT or K308E. Populations of GFP-positive cells arising from homologous recombination were determined by flow cytometry 48 hours post-transfection. Percentages of GFP positive cells arising from HR as measured by flow cytometry are shown as: Con-0.0%, LT- 0.0%, K308E- 0.0%, Sce1- 3.9%, Sce1 + LT- 1.3%, Sce1+ K308E- 3.6%. A representative of four experiments is shown.

First, the interaction of full-length polyoma LT with RPA was demonstrated. LT was immunoprecipitated using antibody to RPA70, and RPA was brought down by antibody to LT (Fig. 5A). The RPA heterotrimer has subunits of 70 (RPA70), 32 (RPA32) and 14 kDa (RPA14) [35]. The small 14 kDa subunit was not found in the LT complex. This result is surprising, since RPA14 and RPA32 form a subcomplex. Most tellingly, LT mutant P402R/G403D (PGRD), defective in transcriptional activation and LT mutant S306P/V358A, which is defective in both specific and non-specific DNA binding showed wild type RPA binding (Fig. 5B). Sequence comparison showed that R154, an SV40 residue critical for RPA binding [38] was conserved between SV40 and polyoma. The comparable polyoma residue, K308, was converted to glutamate. Mutant K308E failed to bind RPA (Fig. 5B).

Cell lines expressing K308E were not sensitive to DNA damage. They did not show drastic morphological changes upon UV treatment (Fig. 5C). A second mutant defective in RPA binding (E320A) was identified (Fig. 5B); it also did not cause increased DNA damage (Fig. 5C). Comet assay results confirmed that the RPA binding mutant K308E fails to enhance the DNA damage response (Fig. 5D), suggesting that abrogation of the interaction of LT with RPA might be able to disrupt LT's ability to increase the DDR in the host cell.

To confirm that DNA repair processes requiring RPA were disrupted by LT, repair of double-strand breaks by homology directed repair was tested [39]. DR-U2OS cells were transfected with I-SceI to generate a double-strand break, and repair was measured by the recovery of intact GFP from two non-functional molecules. By flow cytometry 3.9% of the control cells showed recombination resulting in expression of GFP (Fig. 5E). Only 1.3% of cells cotransfected with WT LT showed recombination, while 3.6% of cells cotransfected with K308E were GFP positive. This shows that LT interfered with homology-directed repair in an RPA-dependent manner.

**Figure 6:**
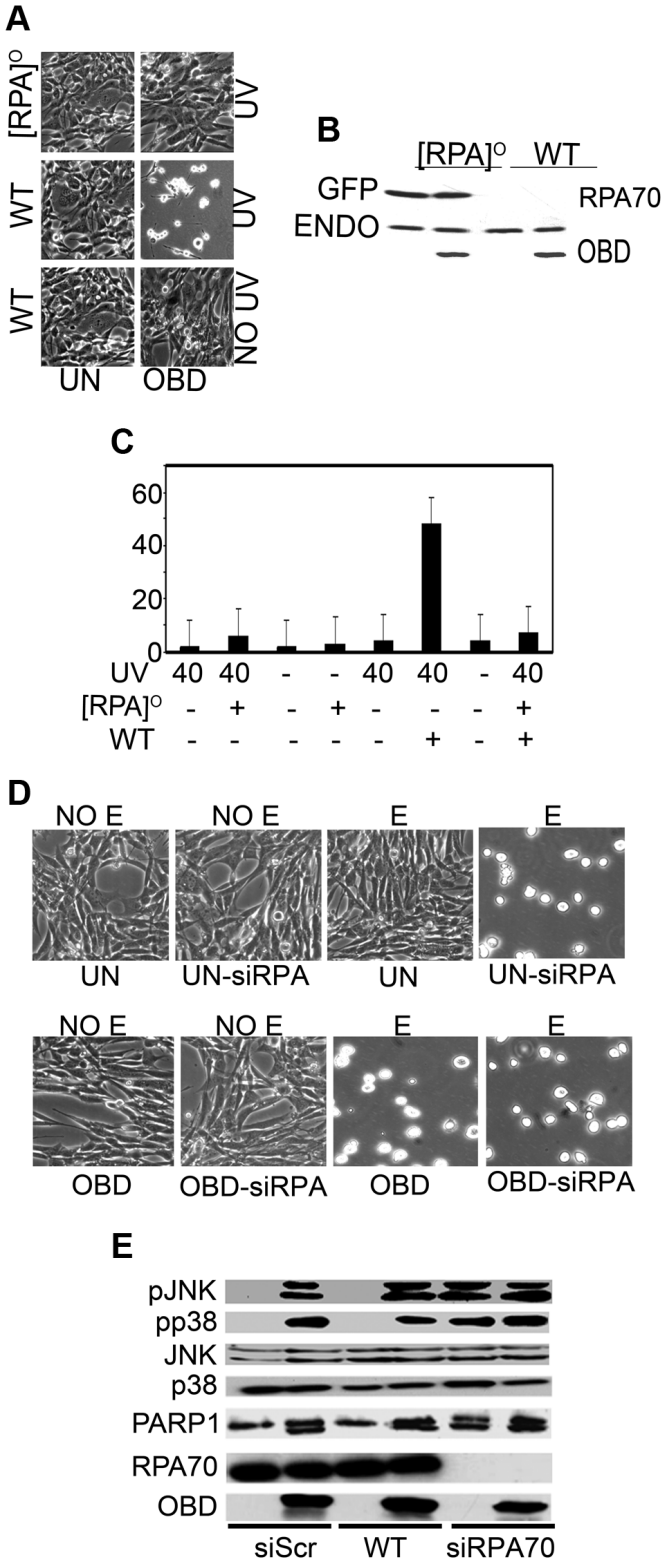
Overexpression of RPA protects against sensitization to DNA damage by LT, while knockdown of RPA mimics LT phenotype. A : Stable, MEF cell lines that inducibly express OBD were used to obtain cells overexpressing RPA using GFP-tagged RPA70 ([RPA]^O^). Morphology of uninduced cells (UN) and cells expressing OBD is shown 16 h after exposure to UV light (40 J/m^2^). **B**: Cell extracts of MEFs inducibly expressing OBD or expressing OBD as well as GFP-RPA were tested by western blot for endogenous (ENDO) and GFP-RPA (GFP) with an anti-RPA70 antibody. **C**: CASP calculated tail moments (TM) from analysis of comet assays of uninduced or OBD-expressing (WT) cells with or without exogenous GFP-RPA70 overexpression either without UV treatment or immediately after UV (40 J/m^2^). Data are shown for a representative experiment, where at least 100 comets were quantitated for each cell line. **D**. Morphology of cells in which RPA has been knocked down. Stable, MEF cell lines that inducibly express OBD were used to obtain cells in which RPA has been transiently knocked down using siRNA directed towards RPA70. Morphology of uninduced cells (UN) and cells expressing OBD is shown 16 h after exposure to 100 µM etoposide (E). Scrambled siRNA (siScr) expressing uninduced (UN) and OBD expressing MEFs was used as negative control. **E**. Uninduced MEFs (UN) or MEFs expressing OBD with transient knockdown of RPA70 (siRPA70) or without (CON) (siScr) were exposed to 40 J/m^2^ UV light. Cell extracts harvested 1 h post treatment were resolved by SDS PAGE and tested by western blotting for PARP-1, phospho-JNK1/2 (pJNK), phospho-p38 (pp38), total JNK1/2 (JNK), endogenous RPA70, total p38 and OBD.

To confirm the role of RPA in sensitization of cells expressing polyoma LT following exposure to DNA damaging agents, we generated stable MEF cell lines that inducibly expressed wild type OBD and simultaneously overexpressed GFP-tagged RPA. Overexpression of RPA about three times higher than the endogenous level protected cells against DNA damage triggered by UV (Fig. 6A & B). Unlike cells that express OBD alone, cells that also overexpress RPA did not show the characteristic increase in comet tail moments in their DNA (Fig. 6C). A final test of the hypothesis that effects on RPA were central to LT sensitization was made by transient RPA70 knockdown. Transient knockdown of RPA70, like LT expression, is accompanied by sensitization to DNA damage (Fig. 6D) and activation of stress responses (Fig. 6E).

### LT Alters Localization of Repair Proteins after DNA Damage through Its Effects on RPA

**Figure 7:**
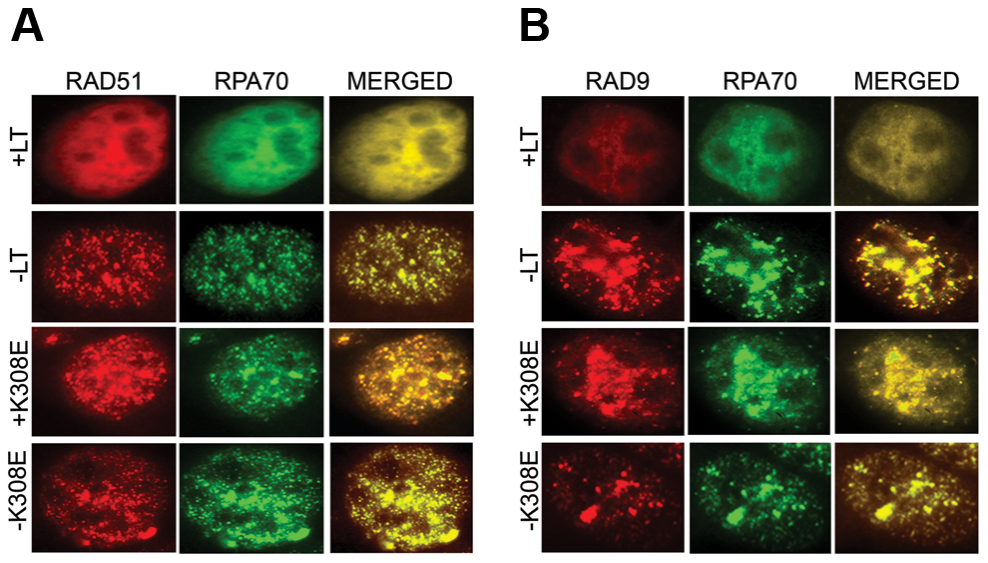
LT inhibits localization of repair proteins into nuclear foci after UV exposure. A : Uninduced MEFs or MEFs expressing either LT or K308E were exposed to 40 J/m^2^ UV. After one hour, they were stained with antibody to RPA70 (FITC), RAD51 (TRITC). Individual fluorescence images and the merged DAPI images that stain nuclei are shown. **B**: Uninduced MEFs or MEFs expressing either LT or K308E were exposed to 40 J/m^2^ UV. After one hour, they were stained with antibody to RPA70 (FITC), RAD9 (TRITC). ). Individual fluorescence images and the merged DAPI images that stain nuclei are shown.

Examination of LT effects on RPA localization provided clue to the problems in DNA repair. After DNA damage RPA is recruited to nuclear sites of damage repair seen as foci [40]. In Fig. 7 it is clear that when LT is expressed, RPA is diffusely nuclear, rather than localizing to the damage foci. Rad51 is critical for homologous recombination [41]. As a result of LT expression, Rad51 is also not recruited to foci after damage, explaining the defect in homologous recombination. The Rad9/Rad1/Hus1 (9-1-1) complex is a sliding clamp important for DNA repair [42]. Like Rad51, Rad9 is prevented from reaching damage foci by LT. The RPA binding mutant K308E had no effect on localization of either Rad51 or Rad9.

## Discussion

These results point to a novel connection between DNA viruses and DNA damage regulation. LT sensitizes cells as much as one-hundred fold to DNA damage from UV irradiation or etoposide. The effect of LT is somewhat reminiscent of past reports of SV40 LT and bleomycin-induced spontaneous DNA damage [29]. LT does not modulate initial DNA damage as measured by the formation of photoproducts after UV, but rather interferes with repair. The result is excessive DNA damage revealed by the comet assays leading to apoptosis. The only unusual feature of LT induced death is the stabilization of Bim and its translocation to the nucleus. This has been seen before with Human Herpes Virus-8, which uses nuclear translocation of Bim to inhibit its activity [43]. The importance of this observation is unclear, because knockdown of Bim had no effect on phenotype. However, it remains possible that more than one member of the BH3 family is perturbed to cause the phenotype.

Genetic studies and biochemical analysis identified the single-stranded DNA binding protein RPA as the target bound by LT to produce sensitivity. Here we have shown that LT through its OBD binds RPA. LT-RPA complexes differ from the endogenous complex in that the 14 kDa RPA3 subunit is lacking. This is somewhat reminiscent of the PyST/PyMT interactions with heterotrimeric protein phosphatase 2A (PP2A), where the PP2A A and C subunits are found in the T antigen complexes, but the B subunit is missing [44]. SV40LT is reported to bind RPA70 constructs that contain DNA binding domains A and B [38]. PyLT also binds an RPA70 A/B construct expressed in E. coli (not shown). The simplest interpretation is that there is an LT-RPA70-RPA32 complex. There may be additional interactions or steric hindrance with the heterotrimer in addition to the A/B interaction that prevent RPA14 association. However, given that RPA14 seems to form a structural core with the DNA binding domain C of RPA70 and DNA binding domain D of RPA32, it is very surprising that RPA14 is missing. It raises a possibility that there are separate LT-RPA70 and LT-RPA32 dimeric complexes. In any case, it is hardly surprising that complexes lacking RPA14 seems to be non-functional in DNA repair. The interaction of RPA with SV40 LT has also been shown to perturb processivity of DNA polymerase α, so it may have effects in replication as well [45]. We have identified two LT mutants, K308E and E320A, which fail to bind RPA and fail to sensitize cells. Both K308E and E320A can activate E2F-containing promoters (not shown), indicating that they retain LT function towards the Rb family. Other functions of OBD, including DNA binding and transcriptional activation, were not required for RPA binding or sensitization. That RPA is the relevant target was confirmed by the demonstration that overexpression of RPA protected from LT and knockdown of RPA mimicked the LT phenotype.

RPA is a protein important for DNA replication and DNA repair [11], [35], [46]. It is required for SV40 DNA replication [38], [47], [48] and it functions for polyoma as well [21]. It is also a common molecular component of most repair mechanisms (see [11] for a recent review). In particular, RPA is a sensor of UV induced DNA damage that is required for repair of the lesions [14], [16]. In addition, RPA participates in the formation of repair foci in response to etoposide-induced double-stranded DNA breaks (DSBs) [13]. LT binding prevents RPA from localizing to sites of DNA damage. This means that DNA damage sites that would normally be occupied by RPA after the DNA insult lack RPA required to trigger efficient removal of the DNA lesions. Thus Rad51 and Rad9 are not recruited to damage foci when LT is expressed. This would account for the observed failure to repair double-strand breaks by homologous recombination when LT was expressed. All of these observations suggest that titration of RPA by LT, pushing in the direction of its replicative functions and away from its repair functions, is the basis for our effect.

In summary, our results demonstrate that interaction of LT with RPA is the pivotal contributing factor to sensitization to DNA damaging agents. It suggests that targeting RPA function might be a useful way to regulate survival. LT-mediated inhibition of RPA can provide a vital strategy in overcoming chemotherapeutic drug resistance and therefore for the treatment of cancer. Inhibition of RPA has in fact been considered as a therapeutic [49]. In the polyomavirus field, Merkel Cell Polyomavirus (MCV) is thought to be responsible for a class of human skin cancers [50]. Although the DNA-binding domain is eventually deleted in MCV tumors, it could easily be imagined that a pro-mutagenic phenotype promoted by MCV LT might contribute to the early progression in such cancers.

## Materials and Methods

Antibodies against p38, phospho p38, JNK, phospho- JNK, PARP-1, BAD, BclXL, Bim, were from Cell Signaling Technology. RPA, Rad51 and Rad9 were from Santa Cruz. Anti-GFP was from Sigma. PN116 monoclonal antibody that recognizes the N-terminal domain was used to detect polyoma large T and its mutants. Anti-HA11 from Covance was used to detect HA-tagged OBD. FITC and TRITC antibody and secondary antibodies were from Jackson Immunochemicals.

### Cell Lines

NIH 3T3 cells were grown in Dulbecco's modified Eagle's medium (DMEM) supplemented with 10% calf serum. Tet-off regulated mouse embryonic fibroblasts (MEFs) that contain the pBI-G Tet-off vector (Clontech) expressing LT antigen, its mutants and the OBD were obtained by selection in 5 µg/ml puromycin after cotransfection with a vector for puromycin resistance and the relevant LT construct. To exclude clonal variation we have analyzed at least six each of full-length and OBD expressing clones. HEK 293T cells were grown in DMEM with 10% fetal calf serum.

### Plasmids and Transfection

pCMVLT, HA-tagged Origin binding domain of PyLT(residues 264 to 420) were previously described [34]. All LT mutations were introduced into pBI-G LT or pCMV LT using site-directed mutagenesis and verified by sequencing.

### RNA Knockdown Analysis

MISSION shRNA clones from Sigma-Aldrich that are sequence-verified shRNA lentiviral plasmids were tested for maximum gene silencing effects. The target sequences for Bim were selected and synthesized by Sigma-Aldrich (NM_009754). Self-inactivating replication incompetent viral particles were produced in packaging cells (HEK293T) by co-transfection with compatible packaging plasmids. The targeting sequence used for Bim that achieved maximum knockdown as measured by immunoblot analysis was purchased from Sigma Aldrich: TRCN0000009694. For siRNA mediated transient knockdown of RPA70, RNA duplexes used for targeting mouse RPA70 were purchased from Qiagen (Gaithersburg, MD; GS68275). The small interfering RNAs (siRNAs) were introduced in cells using Lipofectamine RNAiMAX reagent (Invitrogen) by reverse transfection according to the manufacturer's protocol at a final total concentration of 20 nM. Non-targeting negative control siRNA used was from Qiagen (1027310). After 48 hours, medium was collected and whole cell extracts prepared that were subjected to immunoblotting analysis as described below.

### Immunoprecipitation

Cells were washed with cold PBS, harvested and resuspended in lysis buffer (20 mM Tris, pH 7.5; 150 mM NaCl, 1 mM EGTA, 1 mM EDTA, 1% Triton X-100, 2.5 mM sodium pyrophosphate, 1 mM β-glycerolphosphate, 1 mM Na_3_VO_4_, in the presence of protease inhibitors (1 µg/ml leupeptin, pepstatin, and aprotinin) and phosphatase inhibitors I and II (1∶100; Sigma) for 30 min. Cleared extracts were incubated with specific antibody and protein G Sepharose beads (Amersham) for 4 hours, with rocking, at 4°C. Cell extracts were boiled directly in SDS dissociation buffer. After electrophoresis, samples were blotted onto nitrocellulose and analyzed by immunoblotting [34].

### Immunofluorescence

Cells on glass coverslips were fixed with cold methanol (−20°C) for 20 min at room temperature and washed again three times for 5 min each with TBS (50 mM Tris-HCl, pH 7.4, 150 mM NaCl). Cells were quenched in fresh 0.1% sodium borohydride in TBS for 5 min, washed three times for 5 min each, and blocked with blocking buffer (10% goat serum, 1% BSA, 0.02% NaN_3_, in TBS) for 1 hour at room temperature. Fixed cells were then incubated with primary antibody in blocking buffer overnight at 4°C (1∶100). Cells were again washed three times for 5 minutes each with TBS, followed by incubation with Trit-C or Fit-C labeled secondary antibody at 1∶800 in 1% TBS for one hour at room temperature. Cells were washed again three times for 5 min each and mounted on glass slides with Vectashield (Vector Laboratories) that stains the nuclei. Cells were observed by fluorescence microscopy. Images were captured using a Spot advanced imaging system.

### DNA Laddering

MEF cells were grown in 100 mm plates, chilled on ice for 15 minutes, collected by scraping and centrifugation, washed once with cold PBS, and lysed in 0.4 ml of lysis buffer (10 mM Tris, pH 7.4, 25 mM EDTA, PEG 5000 2.5%, 1M NaCl and 0.25% Triton X-100) on ice for 30 minutes. This was followed by centrifugation at 13,800×g for 15 minutes, and the supernatant was treated with RNase A (200 µg/ml) at 37°C for 1–2 h, followed by incubation with Proteinase K (100 µg/ml) at 56°C overnight. The mixture was then purified sequentially with phenol-chloroform and chloroform and then precipitated with 0.1 volume of 5M NaCl and 2 volumes of ethanol at −20°C overnight. After resuspension, equal amounts of the DNA (determined by spectrometry at 260/280 nm) were loaded on a 2% agarose gel (50 volts for 2 hours), stained with ethidium bromide (1 µg/ml), and observed by UV illuminator.

### Comet Assay

DNA damage in mouse embryonic fibroblast lines was determined under alkaline conditions using the Comet Assay kit from Trevigen (Gaithersburg, MD). Briefly, the cells were trypsinized, washed in ice-cold PBS, combined with molten agarose, and pipetted onto a comet slide. After solidification at 4°C for 20 min, the slides were immersed in lysis solution. For single cell electrophoresis (detects single and double strand DNA breaks, DNA cross-links, and base damage), the slides were placed in alkaline buffer and electrophoresed at 20 volts for 20 minutes at 4°C. Slides were then washed 2 times consecutively for 10 minutes each with H_2_O followed by 70% ethanol for 5 minutes. Air-dried slides were then stained with SYBR green I and analyzed using a fluorescence microscope. Cells with damaged DNA display streaming of DNA fragments from nucleus in the form of a comet tail, whereas undamaged DNA appears in the form of a nucleus). Comet images were analyzed using CASP software (Comet Assay Software Project 1.2.2). At least 100 comets were analyzed for each sample. Comet assays were performed three times, each time in duplicate.

### Infection

NG59RA viral suspension was sonicated and then incubated at 37°C for 20 minutes. Secondary mouse embryo fibroblasts in 100-mm dishes were infected with 2 ml of the viral suspension after washing. Following adsorption for 2 hours at 37°C, cultures were further incubated in fresh medium containing 15% fetal calf serum. Control cultures were mock-infected under identical conditions, but without virus.

### Flow Cytometry Based Determination of the Formation of CPD and 64PP-UV Photolesions

Stable MEF cells expressing LT or OBD under inducible conditions for 48 h after splitting were allowed to grow to 95% confluence until the day of harvest prior to UV treatment. Cell monolayers were washed twice with 2 ml PBS and irradiated with 40 J/m^2^ UV using a UV Stratalinker 2400 (Stratagene). At various times post-UV, cells were washed with PBS plus 50 mM EDTA, trypsinized, resuspended in 1 ml of PBS plus 50 mM EDTA, and fixed by the addition of 3 ml of ice-cold 100% ethanol added dropwise.1×10^6^ fixed cells were then washed with PBS plus 50 mM EDTA, resuspended in either 0.5% Triton X-100 plus 0.1N HCl (for 6-4 photoproduct (6-4PP) detection) or 0.5% Triton-X 100 plus 2N HCl (for CPD detection), and incubated for 20 minutes at 22°C. Cells were washed with 0.1M Na_2_B_4_O_7_ (pH 9.0) and then with PBS and resuspended in 300 µl of RNase (100 µg/ml in PBS) for 1 h at 37°C followed by washing with PBS-TB (1% bovine serum albumin plus 0.25% Tween 20 in PBS). Cells were resuspended in PBS-TB containing a primary monoclonal antibody against either CPD or 6-4PP (Kamiya Biomedical Company) for 1 hour at room temperature. Pellets were washed twice with PBS-TB and resuspended in 300 µl of fluorescein isothiocyanate-conjugated rabbit anti-mouse secondary antibody for 45 minutes at room temperature. Pellets were washed twice with PBS-TB. Samples were then subjected to flow cytometry and analyzed by WinList 3D.

### HR Reporter Assay

The efficiency of homology-directed recombination repair was evaluated using the DR-GFP recombination reporter construct that contains two mutated, non-functional copies of a GFP gene with an 18 base pair I-SceI recognition site. Double-strand breaks induced in the chromosomally integrated GFP gene with the expression of the I-SceI endonuclease was repaired by homologous recombination restoring the expression of the intact functional GFP gene. DR-U2OS cells were transfected with control, LT or K308E with or without cotransfection of I-SceI expression vector for 48 h. Cells were processed for flow cytometry. GFP expression in gated live populations was analyzed using Summit 4.3 Software.

## Supporting Information

Figure S1 **Inducible expression of LT and its mutants in MEFs.** pBI-G MEFs were transduced to give stable cell lines that could express OBD, wild type LT or various LT mutants. Cell extracts were made from uninduced (UN) or cells induced (IND) for 48 h by the absence of doxycline. LT expression from half of a 100 mm dish was determined by western blotting with anti-T antibody, while OBD expression was monitored with HA antibody. Single mutants are named by the amino acid changes. S306P/V358A is shown as S/V, P402R/G403D is PGRD, and the double mutant E343K, E344K is labeled 343KK.
(TIF)
