# Supplementary figures and images for: Viral Interference with DNA Repair by Targeting of the Single-Stranded DNA Binding Protein RPA
Source: PLoS Pathog. 2013 Oct 24;9(10):e1003725. doi: 10.1371/journal.ppat.1003725 (PMC3812037; doi:10.1371/journal.ppat.1003725)

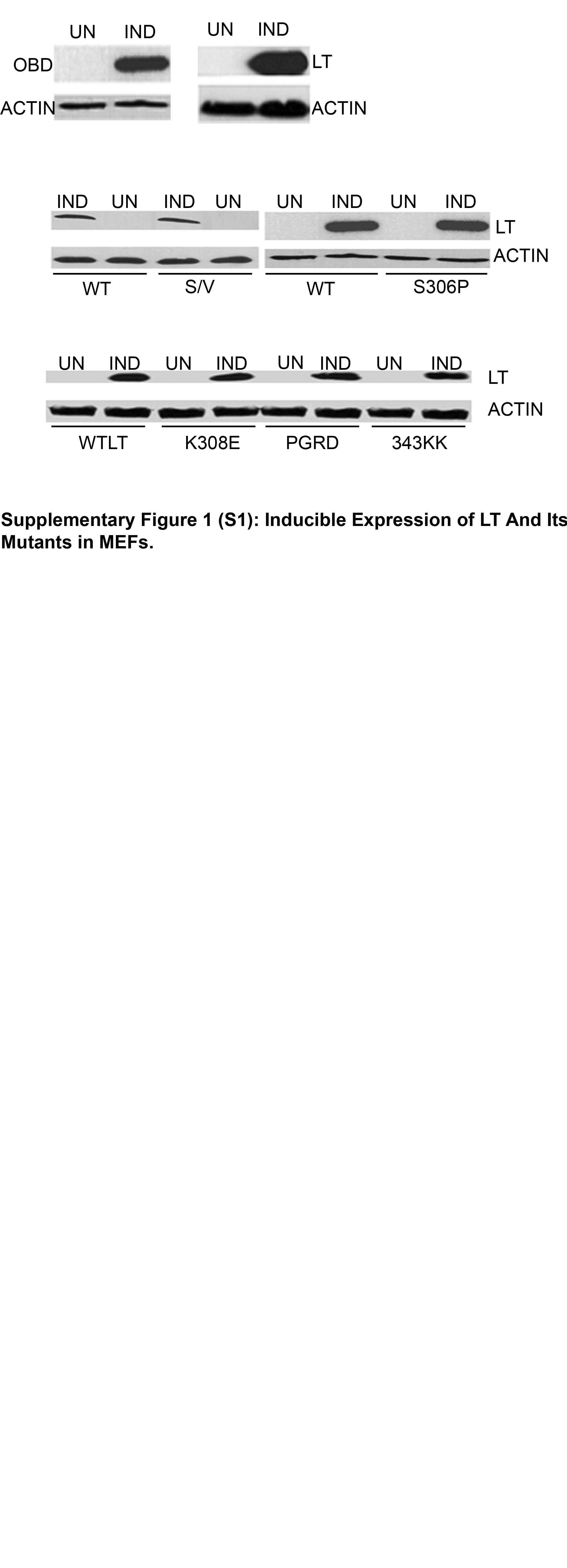

Supplement: Figure S1 — Inducible expression of LT and its mutants in MEFs. pBI-G MEFs were transduced to give stable cell lines that could express OBD, wild type LT or various LT mutants. Cell extracts were made from uninduced (UN) or cells induced (IND) for 48 h by the absence of doxycline. LT expression from half of a 100 mm dish was determined by western blotting with anti-T antibody, while OBD expression was monitored with HA antibody. Single mutants are named by the amino acid changes. S306P/V358A is shown as S/V, P402R/G403D is PGRD, and the double mutant E343K, E344K is labeled 343KK. (TIF) [file ppat.1003725.s001.tif]
